# Continuous High‐Throughput Fabrication of Architected Micromaterials via In‐Air Photopolymerization

**DOI:** 10.1002/adma.202006336

**Published:** 2020-12-04

**Authors:** Jieke Jiang, Gary Shea, Prasansha Rastogi, Tom Kamperman, Cornelis H. Venner, Claas Willem Visser

**Affiliations:** ^1^ Engineering Fluid Dynamics group Department of Thermal and Fluid Engineering Faculty of Engineering Technology University of Twente Enschede 7500AE The Netherlands; ^2^ Department of Developmental BioEngineering Faculty of Science and Technology Technical Medical Centre University of Twente Enschede 7500AE The Netherlands; ^3^ Division of Engineering in Medicine Brigham and Women's Hospital Harvard Medical School Cambridge MA 02139 USA

**Keywords:** in‐air photopolymerization, liquid jets, microfibers, microparticles, shape control

## Abstract

Recent advances in optical coding, drug delivery, diagnostics, tissue engineering, shear‐induced gelation, and functionally engineered rheology crucially depend on microparticles and microfibers with tunable shape, size, and composition. However, scalable manufacturing of the required complex micromaterials remains a long‐standing challenge. Here in‐air polymerization of liquid jets is demonstrated as a novel platform to produce microparticles and microfibers with tunable size, shape, and composition at high throughput (>100 mL h^−1^ per nozzle). The polymerization kinetics is quantitatively investigated and modeled as a function of the ink composition, the UV light intensity, and the velocity of the liquid jet, enabling engineering of complex micromaterials in jetting regimes. The size, morphology, and local chemistry of micromaterials are independently controlled, as highlighted by producing micromaterials using 5 different photopolymers as well as multi‐material composites. Simultaneous optimization of these control parameters yields rapid fabrication of stimuli‐responsive Janus fibers that function as soft actuators. Finally, in‐air photopolymerization enables control over the curvature of printed droplets, as highlighted by high‐throughput printing of microlenses with tunable focal distance. The combination of rapid processing and tunability in composition and architecture opens a new route toward applications of tailored micromaterials in soft matter, medicine, pharmacy, and optics.

## Graphical abstract

In‐air polymerization of a liquid jet is presented as a novel platform to produce tunable micromaterials at flow rates >1 mL min^‐1^ per nozzle. A library of microparticles with tunable size, shape, morphology, and chemistry is presented. Stimuli‐responsive Janus fiber with tunable composition and printed microlens arrays with controllable three‐dimensional curvatures are demonstrated.

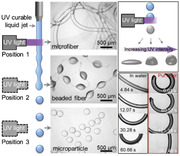

Emerging functional material classes including designer matter^[^ ^1^ ^]^ and bio‐inspired materials^[^ ^2^ ^]^ rely on the availability of meso‐sized building blocks (between the molecular scale and the size of the aimed material) in large quantities. Especially, building blocks on the micro‐scale pose a major opportunity, since they exhibit highly desired features comparable to functional structures in nature and the cellular architecture of our body.^[^ ^3^ ^]^ For example, bio‐inspired microfibers exhibit strong adhesion to human skin,^[^ ^4^ ^]^ microneedle patches enable painless injection,^[^ ^5^ ^]^ microlens arrays inspired by insect achieve improved optical performance,^[^ ^6^ ^]^ and smart textiles adaptively regulate body temperature.^[^ ^7^ ^]^ Microparticles with tunable size and shape were found to prolong blood circulation and enhance vaccine delivery efficacy.^[^ ^8^ ^]^ However, the required massive availability of controllable microparticles can hardly be realized at practically relevant scales.^[^ ^9^ ^]^ Wet chemistry methods for synthesizing nanomaterials are less effective to manufacture fully tailored microparticles that enable these functional features. Methods including emulsion polymerization and electrospinning are scalable but lack precise tunability of the shape and multi‐material composition of monodisperse particles and fibers.^[^ ^10^, ^11^ ^]^ Direct photolithography^[^ ^12^ ^]^ and soft templating^[^ ^13^ ^]^ methods including particle replication in nonwetting templates (PRINT)^[^ ^14^ ^]^ offer precise shape controllability, but lithography setups as well as chemical etching steps are required to fabricate the template. Chip‐based microfluidics^[^ ^15^ ^]^ provides a low‐cost and template‐free method to produce controllable particles with reliable tunability on particle composition and diameter but has limitations in controlling the particle shape. As an advanced chip‐based microfluidic method, stop‐flow lithography^[^ ^16^ ^]^ can produce particles with precisely defined shape but are compromised by their low throughput even after parallelization.^[^ ^17^ ^]^ Continuous‐flow lithography^[^ ^18^ ^]^ has been developed to increase the throughput and, recently, a significant advance^[^ ^19^ ^]^ was made to improve the throughput of microfluidics by operating its flows at jetting regime. Still, the throughput of chip‐based microfluidics is limited to µL min^−1^ scale per nozzle and its parallelization is challenging for complex particles.^[^ ^20^ ^]^ In‐air microfluidics^[^ ^21^ ^]^ exploits liquid jets into air to produce controlled microparticles, at per‐nozzle throughputs that exceed microfluidic chips by typically two orders of magnitude.^[^ ^22^ ^]^ However, this platform is limited to soft hydrogels and hence mainly applicable to tissue engineering applications.^[^ ^23^, ^24^ ^]^ Altogether, the ability to fabricate tailored microparticles in large quantities would address a long‐standing yet urgent fabrication challenge.^[^ ^10^, ^20^, ^25^, ^26^ ^]^

Here we establish a high‐throughput platform to produce micromaterials with controllable aspect‐ratio, outer shape, swelling properties, and stiffness by photo‐crosslinking a liquid jet in air. The polymerization kinetics of the jet is systematically investigated as a function of the exposure time, photoinitiator (PI) concentration, light intensity, and jet diameter. We predicted the local polymerization of the liquid jet and optimized the in‐air processing, enabling the production of a library of micromaterials with tunable shapes, sizes, and compositions. The versatility of in‐air photopolymerization is highlighted by manufacturing mechanically controlled materials, micro‐actuators, and tunable microlens arrays.

As shown in **Figure** **1**a, a liquid jet of UV‐curable polymer was ejected from a nozzle and broke into droplets by the Rayleigh–Plateau instability.^[^ ^27^ ^]^ The droplets were size‐controlled by vibrating the nozzle with a piezoelectric actuator.^[^ ^21^ ^]^ Spatiotemporally controlled photopolymerization of the jet was exploited to control the micromaterials’ shape.^[^ ^28^ ^]^ Since the jet crosses the irradiated area within a few milliseconds (discussed below), a powerful UV light source with adjustable location was focused on specific sections of the polymerizable liquid stream (Figure 1b; for details of the setup in Figure S1, Supporting Information). The laminar jets exhibited steady flow in space and time, and its position was controlled by a manually operated XYZ stage with ≈10 µm precision (see Preparation of the setup in the Supporting Information), enabling its alignment to the light source. Poly(ethylene glycol) diacrylate (PEGDA)‐based inks were used as the model material to demonstrate this approach, as it has well‐known photopolymerizing dynamics and is extensively used in tissue engineering, microneedle patches, printed foams, microlenses, and microfibers.^[^ ^29^ ^]^ Indeed, UV‐exposure of the stable jet resulted in straight fibers that were collected in a downstream liquid bath, as shown in Figure 1c. Similarly, UV‐exposure of the unstable jet or the monodisperse droplets resulted in beaded fibers (Figure 1d) or monodisperse particles (Figure 1e), respectively. These results confirm that UV‐induced polymerization was sufficiently fast for in situ solidification of the in‐air liquid templates.

**Figure 1:**
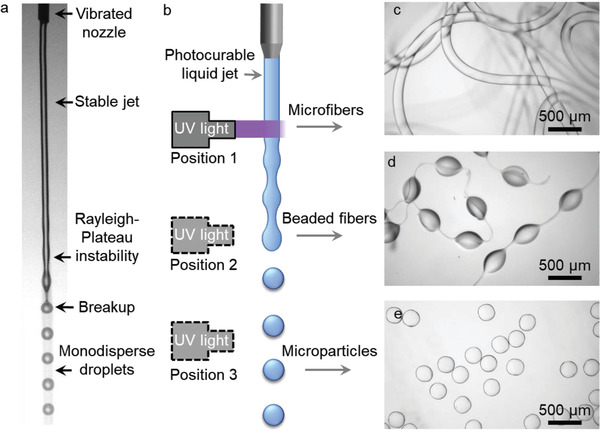
Microfibers and microparticles produced by in‐air photopolymerization of liquid jet. a) Photograph of a photocurable jet (50 wt% PEGDA in water jetting through a nozzle with 150 µm inner diameter), exhibiting a stable jet region, Rayleigh–Plateau unstable region and breakup of monodisperse droplets. b) Scheme illustrating that the photocurable jet is polymerized by UV light, forming continuous fibers, beaded fibers, or particles by adjusting the position of irradiation window. Microscope images of c) straight microfibers, d) beads‐on‐string fibers, and e) monodisperse spherical microparticles.

To unlock the full potential and even predict the outcomes of the microfabrication process, we quantitatively investigate the dynamics of in‐air photopolymerization. **Figure** **2** demonstrates the principles to design this fast‐curing system. In Figure 2a, a representative stable jet of the photocurable PEGDA ink is shown. This stable jet region only occurs for sufficient jet velocities *V*_j_ = 4*Q*/(*πD*_j_^2^), where *Q* and *D*_j_ denote the flow rate and the jet diameter (or nozzle size), respectively. At low ejection velocities, the ink formed large droplets dripping off from the nozzle. The dripping‐to‐jetting transition occurs at Weber number *We* = *ρD*_j_ *V* ^2^/σ ≈ 4, where ρ and σ denote the density and surface tension of the ink, respectively.^[^ ^30^ ^]^ This transition was experimentally verified for our ink, as shown by the open and closed circles in Figure 2c (and pictures in Figure S2, Supporting Information). Flow rates corresponding to *We ≈* 20 provided relatively stable jets (Figure 2a,b) and were therefore selected for our experiments, as shown by the orange markers. Solidifying the uninterrupted section of the jet to produce straight fibers was selected as the indication of sufficiently fast curing; delayed curing would result in irregular fibers. UV light was focused into an 8 mm × 8 mm area (Figure 2b), resulting in an exposure time of τ = *l/V*_j_ with *l* = 8 mm, the height of the illumination window. Figure 2c shows the exposure time as a function of *Q* and *D*_j_, revealing a short polymerization window 1 ms < τ < 5 ms for jets with diameters from 100 to 300 µm.

**Figure 2:**
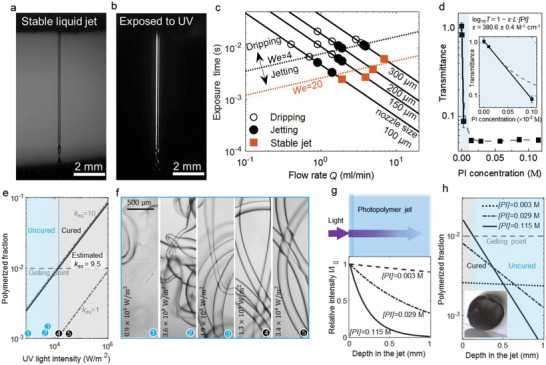
Physical and chemical principles of in‐air photopolymerization. a,b) Photograph showing a jet of PEGDA ink before a) and after b) UV irradiation. The irradiation window has a height of 8 mm. c) The solid lines show calculated UV‐exposure time as a function of the flow rate pass through the irradiation window for different jet diameters. The markers indicate the transition from dripping at low flow rates (open circles) to jetting at increased flow rates (closed circles). The black dotted line indicates the predicted transition Weber number (*We* = 4). Squares indicate the flow rates for stable jetting for each nozzle diameter as used in this work, corresponding to *We* ≈ 20 as indicated. These flow rates correspond to irradiation times in the range of 1 to 5 ms (left axis). d) Measurement of the transmittance (at 375 nm wavelength) of the photoinitiator (PI) as a function of its concentration [PI], providing an absorption coefficient ε = 380.6 ± 0.4 m ^−1^ cm^−1^. The shaded area is magnified in the inset. e) Inclined lines indicate the predicted polymerized fraction as a function of the UV intensity, for different values of the reaction constant, *k*_eq_, as indicated. Solidification is expected for 1% polymerized fraction (gelling point, horizontal dashed line). The markers (bottom) correspond to (f). Matching the solidification threshold (f, black marker numbered 4) to the gelation threshold (horizontal dashed line) by *k*_eq_ provides *k*_eq_ = 9.5. f) Left to right: Microscope images of PEGDA fibers obtained at increasing UV light intensity (*I*_0_), showing consistent results for *I*_0_ ≥ 10^4^ W m^−2^ (black markers). g) Predicted light intensity as a function of the transmission depth (*z*) inside the jet for different [PI], based on our measured ε. h) Calculated polymerized fraction at varying depth of liquid jet (*z*) for different [PI], based on our estimated *k*_eq_. The shades show the cured (gray) and uncured (blue) sections of the jet for [PI] = 0.115 m. The inset shows a hollow fiber, fabricated by selective curing of the surface of the liquid jet.

Polymerization of the jet is initiated at the surface, where the UV intensity is the highest. For chain reactions as prevalent in our PEGDA ink, the polymerized fraction is derived as [1‐exp(‐*k*_eq_(*βε*[*PI*]*I*_0_ *e^−ε[PI]z^*)^1/2^τ)] (see the Supporting Information) with the absorption coefficient of UV light by the ink (ε), the concentration of photoinitiator, ([PI]), the UV light intensity (*I*_0_), the equivalent reaction rate constant (*k*_eq_), the reaction time (τ = 1 ms is used; equals to the exposure time), *z* the depth into the jet (*z* = 0 at the surface), and β a constant (see the Supporting Information). We carefully selected a PI with an absorption spectrum that match the emission wavelength of our UV source (peak at 375 nm, see Figure S3, Supporting Information). The PI concentration was set to 0.115 m (4 wt%) to achieve high UV absorption, which combined with the intense LEDs, provided fast polymerization (Figure S4, Supporting Information). Higher concentrations of PI made the ink very sensitive and resulted in irregular fibers while lower concentrations resulted in curled fibers (Figure S5, Supporting Information). The absorption coefficient of the ink was measured as 380.6 ± 0.4 m ^−1^ cm^−1^ which is similar to reported values^[^ ^31^ ^]^ (Figure 2d). With these input parameters, the polymerized fraction is plotted as a function of the light intensity for different rate constants in Figure 2e. The rate constant was plotted for *k*_eq_ = 1 and *k*_eq_ = 10, corresponding to its expected lower and upper boundaries.^[^ ^32^ ^]^ We assume that shape stability by solidification is reached at the gelation point, occurring at 1% polymerized fraction for PEGDA^[^ ^33^ ^]^ (indicated by horizontal dashed line in Figure 2e). Now, depending on the rate constant, Figure 2e shows that the minimum intensity to solidify the jet at the surface may fall in a very wide range. Therefore, we experimentally obtained the rate constant for our ink. First, we assessed the shape fidelity as a function of the intensity (Figure 2f), revealing straight microfibers for a threshold intensity *I*_0_ ≥ 1.3 × 10^4^ W m^−2^. Second, we combine the required polymerization fraction of 1% (horizontal dashed line in Figure 2e) with the threshold intensity (vertical dotted line), to obtain *k*_eq_ ≈ 9.5. This value is close to the expected upper boundary of *k*_eq_, confirming the fast curing capability of our system.

These predictable solidification dynamics were readily leveraged to fabricate fibers with a tunable morphology. For example, the absorption of UV light is shown as a function of the traveled distance within the jet for different PI concentrations in Figure 2g, resulting in a nontrivial spatial distribution of the polymerized fraction as shown in Figure 2h. If the model is correct, a thick jet with a higher concentration of PI should polymerize only at the surface, yielding a hollow tube. The inset in Figure 2h (also see Figure S6, Supporting Information) shows the hollow tube, thereby validating the model. This example successfully demonstrates the predictive fabrication of complex microstructures by modeling the in‐air photopolymerization kinetics.

In‐air photopolymerization facilitates tremendous tunability of the size, shape, and composition of the resulting microparticles and microfibers, since the jet diameter and shape, the spatiotemporal UV exposure, and the ink composition are independently controlled. Particles and fibers with diameters ranging from 100 to 600 µm were produced by changing the diameter of the nozzle orifice (**Figure** **3**a,b and Figure S7: Supporting Information). Those particles seem to have heterogeneous structures (Figure S8, Supporting Information) induced by the multidirectional UV exposure. As shown in Figure 3c, the particles possess monodisperse size distributions, since rapid polymerization solidifies the monodisperse droplets before they collide. The particles are larger than the nozzle diameter, since break‐up results in droplets with a diameter ≈1.8 *D*_j_. Continuous fibers with tunable and consistent diameter are produced at velocities up to 4.2 m s^−1^, providing an efficient way (e.g., Figure S9, Supporting Information) to print hydrogel scaffolds for biomedical applications.^[^ ^34^ ^]^ The nozzle shape was adjusted to tune the cross‐sectional shape of the fibers. Hollow fibers were achieved by applying a core–shell nozzle that ejected a non‐curable liquid center and a polymerizable liquid coating (Figure 3d). Similarly, a semi‐cylinder was fabricated by placing two parallelly aligned nozzle exits close to each other, resulting in fluid merging^[^ ^24^ ^]^ of one photocurable jet and one non‐curable jet (Figure 3e).

**Figure 3:**
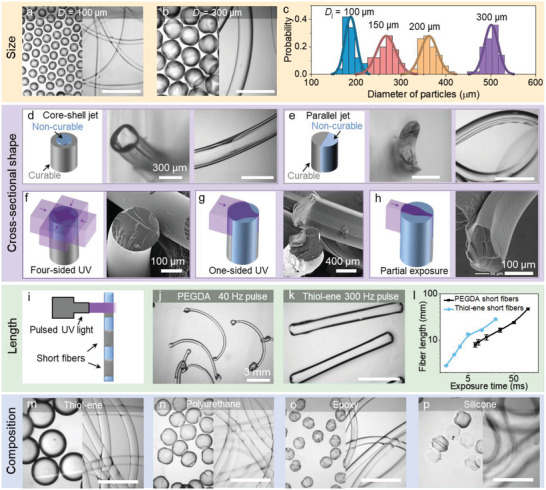
Library of microparticles and microfibers with controlled size, shape, and composition. a,b) Optical microscope images of microparticles and microfibers of PEGDA fabricated from nozzles with inner diameter (*D*_j_) of 100 µm (a) and 300 µm (b). c) The size distributions of PEGDA microparticles. Each statistic is based on 50 measurements. Histograms indicate measured data and solid lines indicate curves of fitting normal distributions to the measured data. d) Scheme and optical microscope images of hollow PEGDA fibers produced from a core–shell nozzle. PI is absent in the core jet to make it non‐curable. e) Scheme and optical microscope images of semicylindrical PEGDA fiber produced from a combined jet of curable liquid and incurable liquid. The liquids were ejected from separate nozzles and merged in‐air before UV exposure. f–h) Schemes and SEM microscope images of microfibers with controlled cross‐section by tuning the number and type of UV light sources. A cylindrical fiber was made by four‐directional UV exposure. Triangle‐shaped fibers were made from one‐sided exposure to a wide UV laser beam. Ribbon‐shaped microfibers were produced from one‐sided partial exposure of liquid jet to a narrow UV laser beam. i) Scheme of the production of short fibers by pulsing the UV light. Optical microscope images showing short fibers of j) PEGDA and k) thiol‐ene achieved by pulsing the UV light. l) Dependence of the length of short fibers on the exposure time to pulsed UV light. m–p) Optical microscope images of microparticles and microfibers produced from thiol‐ene (m), polyurethane (n), epoxy (o), and silicone (p) ‐containing inks (molecular structures in Figure S11, Supporting Information). Scale bars represent 1 mm unless stated otherwise.

Nozzle‐independent shaping of micromaterials was achieved by exposing only a section of the jet to the UV light. For example, cylindrical fibers (Figure 3f) and asymmetric filaments (Figure 3g) were produced by four‐sided irradiation and single‐sided irradiation, respectively. Partial exposure to a thin beam only solidified a ribbon‐shaped section of the jet (Figure 3h). Therefore, our platform enables scalable production of fibers with noncircular cross‐sections, for applications including guiding the growth of cells.^[^ ^35^ ^]^ Fibers with adjustable length were enabled by flashing of the UV light, so that only a section of the jet was polymerized (Figure 3i). In this fashion, PEGDA fibers of arbitrary length were readily fabricated (Figure 3j). However, the fibers became partially connected for a length below 8 mm (Figure S10, Supporting Information), possibly due to spatial overlap of UV‐exposed fiber sections within the 8 mm exposure window. Combining the fast‐curing thiol‐ene inks^[^ ^36^ ^]^ with a smaller irradiation window (1 mm × 1 mm square) permits the production of fibers as short as 2 mm with well‐defined ends (Figure 3k). The measured length of both types of short fibers monotonically increased with the exposure time of pulsed UV light (Figure 3l), demonstrating rapid fabrication of length‐controlled fibers by in‐air photocuring.

Microparticles and fibers were produced in thiol‐ene, polyurethane (PU), epoxy, and silicone‐enhanced inks, as shown in Figure 3m–p (molecular structures in Figure S11 (Supporting Information), FTIR spectroscopy in Figure S12 (Supporting Information)). Tuning the chemistry resulted in a wide range of fibers with tensile strength from 10 to 300 MPa (corresponding to Young's modules from 3.0 to 3.9 × 10^5^ MPa) (Figure S13, Supporting Information), with epoxy providing the strongest and stiffest fibers. Figure S14 (Supporting Information) shows that the swelling ratio is a function of the chemistry and the immersion liquid. A wide range from no swelling (various materials in water, ethanol, or acetone) to 50% extension (PU in acetone and 50% PEGDA in water) was observed. These differences in material types and properties could inspire new applications. For example, the insensitivity of thiol‐ene reactions^[^ ^37^ ^]^ to oxygen and water allows the development of robust lithography platforms, the adjustable ratio of soft and hard molecular segments of polyurethane^[^ ^38^ ^]^ and epoxy enables controlled underwater acoustic absorption and actuation (explored below), and silicone chemistry^[^ ^39^ ^]^ provides excellent biocompatibility. Defects and non‐uniformities were observed for the polyurethane and epoxy particles (Figure 3n,o). The nonuniform structures might result from the added diluent to adjust the ink viscosity (see extended method in the Supporting Information), which can be prevented or tuned by reducing the amount or changing the type of diluent.

Independent tunability of the length, diameter, cross‐section, and chemistry provides exceptional design flexibility of micromaterials since these features can be combined to yield anisotropic micromaterials with complex yet deterministically controlled morphology and composition. Here we demonstrate this ability by fabricating stimuli‐responsive Janus microfibers with controllable composition by in‐air polymerizing a heterogeneous liquid jet. To make the fiber responsive to the stimuli of water, PEGDA and epoxy inks were selected to form the heterogeneous jet based on their distinct swelling ratio in water (Figure S14, Supporting Information). The two aligned liquid jets were merged and polymerized in‐air at the speed of 0.5 m s^−1^ (**Figure** **4**a). After contact with water, a straight fiber bent into a circle (Figure 4b) and recovered to its original straight shape when put back into air (Figure 4c). The curvature of these fibers as a function of time is plotted in Figure 4d, revealing strong deformations with a maximum curvature 1.5 mm^−1^ achieved within ≈1 min. Subsequent relaxation to 0 mm^−1^ in air confirms the capability of fast and reversible deformation. The structure of the responsive fiber kept stable after tens of reversible bending cycles (Movie S1, Supporting Information), indicating the robustness of the Janus fiber. The ratio of two resins in the Janus fiber was adjusted by tuning the flow rate of two jets. Strong tunability of the curling was observed (Figure 4e). The maximal curling was reached when the PEGDA:epoxy thickness ratio was ≈3. This result is close to the calculation based on the Timoshenko model,^[^ ^40^ ^]^ which indicates the bending curvature of the Janus structure reaches the maximum when the thickness ratio of PEGDA and epoxy is around 2 (Figure 4f, details see in the Supporting Information). As a proof‐of‐concept demonstration of the Janus fiber to function as a soft actuator, a small object was lifted by the deforming fiber under water (Figure 4g). The estimated force exerted by the microfiber was 16.5 mN, resulting in stress of 0.1 MPa across the fiber. The object was lifted 4.3 cm which is almost half of its original length (9.0 cm), indicating the as‐produced fiber comprises a fully functional soft actuator. The in‐air polymerization platform provides a highly efficient method to produce stimuli‐responsive fibers with precise controllability for applications including smart textiles where large quantities of responsive fibers are needed.

**Figure 4:**
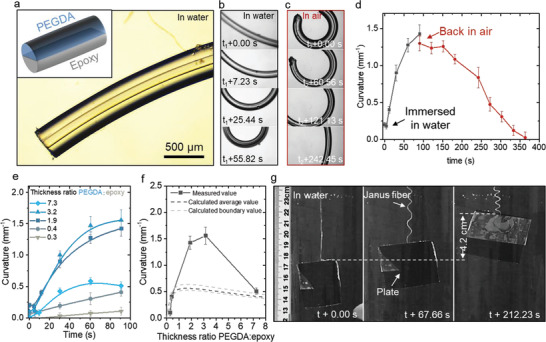
Stimuli‐responsive Janus fibers. a) Microscope image and scheme showing Janus fiber composed of PEGDA and epoxy sections. b) Snapshots of the Janus fiber after exposure to water, showing increasing curvature as a function of time (top to bottom) due to the difference in swelling ratios of PEGDA and epoxy. c) The curved fiber became straight after being placed back in air. d) Time‐dependent curvatures of the fiber in (b,c). e) Measured deformation of fibers with varying ratios of PEGDA and epoxy after contacting with water. The lines are a guide to the eye. f) Measured and calculated value of curvatures as a function of the thickness ratio of two sections. For details of the calculation see the Supporting Information. g) Successive optical images showing a plate being lifted by the in‐air polymerized Janus fiber underwater.

Beyond particle fabrication, in‐air photopolymerization also enables on‐the‐fly control of droplet spreading upon impact. This feat is highly beneficial for additive manufacturing and inkjet printing applications. Here, we demonstrate this ability for ultrafast manufacturing of tunable microlenses onto transparent surfaces. Inspired by compound eyes, microlenses offer enhanced light collimation and collecting efficiency.^[^ ^41^ ^]^ Compared with time‐consuming lithography‐based methods, the readily available ink‐jet printing technique offers an alternative to produce microlenses in one‐step.^[^ ^42^ ^]^ However, the curvature of the printed microlenses is typically determined by the morphologies and wetting patterns of the surface, limiting the material choice. Application of microlens arrays onto solar cells for increased light harvesting^[^ ^43^ ^]^ or onto OLED displays for improved light extraction efficiency^[^ ^44^ ^]^ requires fast, low‐cost, and large‐scale production of patterned microlenses, which cannot easily be met by current lithography methods. Here we developed a high‐speed conformal printing method based on in‐air liquid jet photopolymerization (**Figure** **5**a). Using this method, 4700 microlenses array is produced on a large‐scale (28 cm × 10 cm) soft surface within 5 s by a single nozzle, as is shown in Figure 5b–d. Our method has the unique option to leverage in‐air curing to dramatically increase the viscosity of the droplet prior to impact. Consequently, the spreading of the droplet is reduced,^[^ ^45^ ^]^ providing control over the curvatures of the printed microlenses on homogeneous substrates (Figure 5e). Top‐view microscope images in Figure 5f–h, show the diameters of printed particles decreased from left to right, indicating that inhibition of spreading increased with the UV intensity. Deposition of uncured less viscous ink droplets on the fast‐moving substrate result in microlenses with non‐uniform shapes (Figure 5f). This non‐uniformity is prevented by partial in‐air polymerization and, as a result, microlenses with circular shapes are obtained (Figure 5g,h). Alternatively, matching the tangent velocity of the droplets with the surface velocity of the substrate may prevent non‐circularity, as the droplets would impact perpendicularly as seen from the frame of reference following the substrate. Side‐view images in Figure 5i–k confirmed the formation of 3D morphologies with increasing curvature. Following the scheme in Figure 5l, the focusing ability of the microlens was tested with a simple printed photomask. As shown in Figure 5m–r, the focal distance of the printed microlens can be adjusted in a wide range (0.5–9.0 mm), which is useful in miniaturized optical systems and high‐resolution optical imaging.^[^ ^46^ ^]^

**Figure 5:**
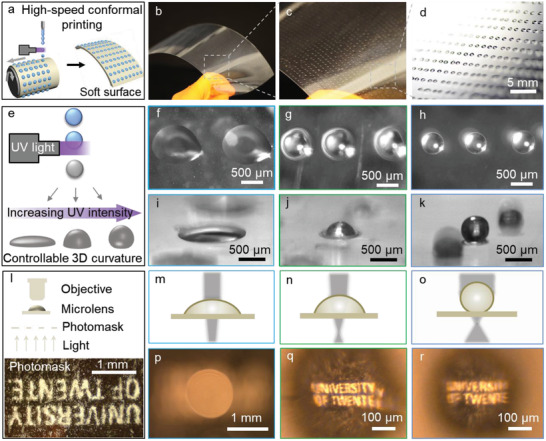
High‐speed conformal printing by liquid jetting and printed microlenses with adjustable 3D curvatures enabled by the in‐air tunability. a) Scheme showing high‐speed conformal printing by jetting microdroplets onto a rotating soft surface. b,c) Photographs of patterned microdroplets array at different magnifications. The entire sheet measures 28 cm × 10 cm and the array contains ≈4700 microlenses that are produced within 5 s by a single nozzle. d) Photograph of cured microdroplets array. e) Scheme showing the curvature of printed particles as controlled by tuning the intensity of UV light. f–k) Microscope images of in‐air tuned microlenses on a glass slide. From left to right, top views (f–h) and side views (i–k) show microlenses with increasing curvatures by increasing the UV intensity. l) Scheme showing the testing system of microlenses. The inset shows a photomask printed on paper used for testing the lenses; the fibers in the paper are clearly visible. m–o) Schemes of sectional view and p–r) captured images of the tested microlens with varying focal distance and field of view. The estimated focal length of microlens based on their radius in (p,q,r) is 9.0, 0.6, and 0.5 mm, respectively. The focal length of microlens in (p) is out of the focusing range of the observing microscope.

In summary, we developed in‐air polymerization of liquid jets as a novel platform to produce microparticles and microfibers with tunable size, shape, morphology, and composition at high throughput (>100 mL h^−1^ per nozzle for all particle sizes). The polymerization kinetics were quantitatively investigated and modeled as a function of the ink composition, the UV light intensity, and the velocity of the liquid jet, enabling engineering of complex micromaterials at high speed. A library of micromaterials with independently tunable diameter, length, morphology, and composition was produced by controlling the nozzle orifice(s), adjusting the irradiating area, pulsing the light source, or modifying the formulation of the ink. The continuous fabrication of complex micromaterials at high‐throughput makes the in‐air photopolymerization platform attractive in providing practically relevant scale of functional building blocks for advanced materials and devices. As the first example, stimuli‐responsive Janus fibers function as soft actuators was produced at the speed up to 0.5 m s^−1^. Furthermore, we demonstrate that in‐air photopolymerization also facilitates precise tuning of printed droplets at high‐throughput, as highlighted by printed microlenses with controllable 3D curvatures. These examples in printed optics and micro‐robotics show that in‐air polymerization of liquid jets establishes a generalizable high‐throughput platform for rapid fabrication of functional micromaterials. Manufacturing of microparticles for optical coding, drug delivery and diagnostics, tissue engineering, shear‐induced gelation, and functionally engineered rheology that depends on particle shape seems feasible.^[^ ^19^, ^25^ ^]^ By alleviating a decades‐old trade‐off between the ability to tailor the particles architecture and throughput, we believe that in‐air photopolymerization provides an effective new strategy toward broad applications of recent and novel microfluidic and particle‐based concepts.

## Experimental Section

### Fabrication of Fibers and Monodisperse Particles from Liquid Jet and Droplets

The inks were ejected from the nozzle at certain flow rate to ensure a stable liquid jet formed. For the fabrication of fibers, flow rates of 2.0, 4.0, 5.0, 7.0, and 11.0 mL min^−1^ were used for nozzles with inner diameter of 100 ± 20, 150 ± 20, 200 ± 20, 300 ± 20, and 500 ± 20 µm, respectively. For the fabrication of particles, flow rates of 1.1, 3.0, 4.4, 6.0, and 9.0 mL min^−1^ were used for nozzles with inner diameter of 100 ± 20, 150 ± 20, 200 ± 20, 300 ± 20, and 500 ± 20 µm, respectively. The UV light was turned on to polymerize the liquid jet. For length‐controlled fibers, the LEDs were pulsed by a function generator. For one‐sided UV exposure and partial UV exposure, the UV laser diode was used as the light source. To generate monodisperse droplets for producing particles, a piezo element was attached to the nozzle to generate vibration in the direction perpendicular to the jet. The piezo element was driven by signal of the sine wave from a function generator and a high voltage amplifier. The piezoelectric element was turned on after the syringe pump was on and the frequency was tuned until a train of monodisperse droplets was visualized by the camera. Typically, frequencies within the range of 0.5 to 3 kHz were applied. After a train of monodisperse droplets was visualized, the UV light was turned on. The fibers and particles were collected at 20 cm distance by a petri dish containing a layer of isopropanol to prevent adhesion.

Further experimental details can be found in the Supporting Information.

## Conflict of Interest

The authors declare no conflict of interest.

## Supporting information

Supporting Information

Supplemental Video 1
