# Supplementary material for: Continuous High‐Throughput Fabrication of Architected Micromaterials via In‐Air Photopolymerization
Source: Adv Mater. 2020 Dec 4;33(3):2006336. doi: 10.1002/adma.202006336 (PMC11468713; doi:10.1002/adma.202006336)
Supplement: Supplementary file 1 — Supporting Information [file ADMA-33-2006336-s001.pdf]

© 2020 Wiley-VCH GmbH

ADVANCED MATERIALS

Supporting Information

for *Adv. Mater.*, DOI: 10.1002/adma.202006336

Continuous High-Throughput Fabrication of Architected Micromaterials via In-Air Photopolymerization

*Jieke Jiang, Gary Shea, Prasansha Rastogi, Tom Kamperman, Cornelis H. Venner, and Claas Willem Visser**

© 2020 Wiley-VCH GmbH

Supporting Information

Continuous high-throughput fabrication of architected micromaterials via in-air photopolymerization

*Jieke Jiang, Gary Shea, Prasansha Rastogi, Tom Kamperman, Cornelis H. Venner, and Claas Willem Visser**

Extended methods

Preparation of the setup

The setup consisted of four LEDs (375 nm, 1270 mW, M375D4, Thorlabs) mounted onto homemade aluminum heat sinks surrounding a nozzle from four directions with 20 cm from LED to liquid jet ejected from the nozzle. LEDs were connected to a 24 V power supply with tunable output voltage to adjust the intensity of UV light. Two condenser lenses are fixed in front of each LED to focus the UV light into 8 mm × 8mm or 1 mm × 1 mm square spot. The UV light intensity was measured by a printed circuit board (PCB)-mounted thermal detector (TD10XP, Thorlabs) connected with an oscilloscope. The UV intensity of the four LEDs that operated at full power and focused into 8mm × 8mm was measured to be 3.4×10^4 W/m^2. Experiments and calculations used this condition unless stated otherwise. Nozzles were made from hypodermic needles (FINE-JECT, Sigma-Aldrich) with measured inner diameter as 100 ± 20 µm (33 gauge), 150 ± 20 µm (30 gauge), 200 ± 20 µm (27 gauge), and 500 ± 20 µm (24 gauge); needles were cut to have a length of around 10 mm length and polished to have flat tips before being used as the nozzles. The nozzles were connected to PEEK tubing using threaded Luer adapter (IDEX). UV light was blocked from the tip of the nozzle to prevent nozzle clogging using polyethylene tubing with an inner diameter of 13.7 mm and length of 50 mm wrapped with heavy-duty black electrical tape (3M) and fitted to the Luer adapter. The tip of the needle was recessed around 5 mm from the end of the opaque covered tubing. The nozzle together with the shelter was mounted to an assembled 3D movable stage (DTS25/M and DTSA03, Thorlabs) to eject a liquid jet vertically downward. A camera (UI-1240LE, IDS) with lens (M118FM25, Tamron) was mounted next to the nozzle and a visible light source was placed opposite to the camera to visualize the jet. A syringe pump with 5 ml steel syringes ((NE800 and SYR-SS5, New Era Pump Systems Inc.) was used to control the flow rate of liquid jet. In experiments that required higher UV intensity or smaller focusing area, a high-power laser diode (RLT405500MG, Roithner LaserTechnik GmbH) mounted onto a PCB with homemade electronics was used as the light source. A collimation lens was mounted in front of the diode for converting the emitted light into a UV beam.

Preparation of UV curable ink.

All the following mixtures were stirred in dark up to 30 minutes until a clear solution was derived. Mixtures except for thiol-ene ink were purged with nitrogen for 10 min before filling syringes to remove oxygen traces that inhibit photopolymerization. For PEGDA particles, 20 ml PEGDA 700 (Sigma-Aldrich) was dissolved into 20 ml deionized (DI) water (Sigma-Aldrich) and 0.4 g lithium phenyl-2,4,6-trimethylbenzoylphosphinate (LAP) was added as PI. To prepare inks for PEGDA fiber, 20 ml PEGDA 700 was mixed with 0.8 g Diphenyl(2,4,6-trimethylbenzoyl)phosphine oxide (TPO) (Sigma-Aldrich). For epoxy particles, 5 ml of an epoxy diacrylate (Actilane 72, Sartomer, Arkema Group) was mixed with 45 ml cyclic trimethylolpropane formal (mono)acrylate (SR531, Sartomer, Arkema Group) and 0.5 g TPO.

For epoxy fiber, 20 ml of Actilane 72 was mixed with 20 ml SR531 and 0.4 g TPO. For polyurethane particles, 5 ml of an aromatic urethane diacrylate (Actilane 20, Sartomer, Arkema Group) was mixed with 45 ml 3-methyl 1,5-pentanediol diacrylate diluent (SR341, Sartomer, Arkema Group) and 0.5 g TPO. For polyurethane particles, 20 ml Actilane 20 was mixed with 20 ml SR341 and 0.5 g TPO. For thiol-ene particles and fibers, 5.95 g of Pentaerythritol tetrakis(3-mercaptopropionate) (PETMP, Sigma-Aldrich), 3.84 g di(ethylene glycol) divinyl ether (DEGDE, Sigma-Aldrich), 0.2 g TPO, and 0.01135 g pyrogallol inhibitor (to stabilize ink in liquid form) were mixed. The as-prepared PEGDA ink was still working properly in the fabrication of continuous fibers after being stored at 5°C for 6 months (purged with nitrogen gas before storage and purged again before use). We expect other types of inks will have similar shelf-time.

Mechanical and swelling measurement of microparticles and microfibers

Tensile measurement of PEGDA microfiber and soft epoxy fiber was performed with an extensometer (Zwick, Z1.0). The fibers with length of 40 mm were clamped to a 100 N force cell for the measurement. Due to the closure of labs, the tensile curve of stiff epoxy fiber (blue curve) is measured by a homemade setup composed with a hanging scale to measure the force and a camera to record the elongation.

To measure the swelling ratio of the in-air crosslinked polymers. Microfibers or microparticles were immersed into solvents or water and were sealed into a vial for 20 hours. The diameter of particles or the length of microfibers were measured before and after the swelling. Swelling ratios were calculated by using the dimension after swelling divided by the corresponding original dimension.

Fabrication and characterization of Janus fibers

Two needles with inner diameter of 500 ± 20 µm were aligned together by paraffin film and were connected with syringe pumps. The flow rate of the two jets was adjusted separately while the total flow rate was kept as 12 ml/min to generate a stable combined jet. The cured fibers were collected in an empty petri dish. Short fiber with a length of around 5 mm was cut from the continuous fiber for the deforming test. The short fiber was put into an empty petri dish and DI water was added. The process of the deformation was recorded by a microscope. A 15 cm fiber is used for the demonstration of lifting objects underwater. The fiber was attached to a rubber plate with dimensions of 48.48 mm × 39.53 mm × 1.67 mm and the entire assembly was gently placed underwater to allow the fiber to deform. Densities of 1522 kg/m^3 for rubber and 997 kg/m^3 for water were used to estimate the sinking force of rubber plate exerted to the fiber. The distance from the water surface to the collecting point of the fiber was measured to be 9.04 cm.

Fabrication and characterization of microlens

Two types of rotating stages were used to produce microlens array. For the large-scale production of microlenses on a soft substrate, a gear motor was attached to a writing robot to achieve both rotating and lateral movement. A soft Grafix clear craft plastic film is rolled onto a cylinder (90 mm diameter) that was driven by the gear motor. The spinning speed of the gear motor was set as 450 RPM and the lateral speed of the writing robot was set as 5 cm/s. For the small-scale production of microlens on glass slides, a homemade spinning stage is used to support the glass slide for collecting the printed microlens. Plain microscope glass slides were cleaned thoroughly by ethanol and dried by compressed air before being used as the collecting substrate. The glass slide was attached to the turning stage with double-sided tape. The turning speed was set as 300 rpm. UV light was turned on after stable monodisperse droplets were visualized from the monitoring camera. In the meantime, the turning substrate

was moved through the jet slowly by hand to collect the particles. All the printed samples were put under the UV light to post-cure up to 30 min to solidify the microlenses.

Calculations

Calculation of polymerized fraction

Our in-air polymerization system is characterized by radical chain photopolymerization, that we model following a previous study[1]. The polymerization rate is used to relate the polymerized fraction of material with experimental parameters including UV light intensity (I_0), time of reaction (t), absorption coefficient (ε) and concentration of photoinitiator, PI, ($[PI]$).

Monomers are consumed by both the initiation and propagation. However, the number of monomer molecules reacting in the initiation step is far less than the number in the propagation step for a process producing polymer. To close approximation, the rate of monomer disappearance, which is synonymous the polymerization rate ($-\frac{d[M]}{dt}$) is given simply by the rate of propagation (r_p).

$$-\frac{d[M]}{dt} = r_p \quad (1)$$

For a general radical chain photopolymerization reaction, the rate of photopolymerization can be related to light intensity by[1]

$$r_p = k_p[M]\left(\frac{\phi\alpha[A]I_0 10^3 e^{-\alpha[A]D}}{k_t}\right)^{\frac{1}{2}} \quad (2)$$

where k_p is the propagation reaction rate constant, ϕ is the PI efficiency, k_t is the termination reaction rate constant, α is the absorption coefficient of PI, [A] is the molar concentration of PI, the light intensity (I_0) is expressed as moles of photons delivered per square centimeter per second (mol·cm^{-2}·s^{-1}), and D is the distance to the surface and has the unit of cm. To adapt the equation into the in-air photopolymerization system, symbols of α, [A] and D are replaced with ε, [PI] and z, respectively, without changing the represented meanings. The I_0 in the specification of our UV light source is expressed as watts per square meter (W/m^2). Therefore, the unit of I_0 in mol·cm^{-2}·s^{-1} (moles of photons delivered per square centimeter per second) is converted to W·m^{-2}, and the unit of D in cm is converted to the unit of z in m. We have

$$r_p = k_{eq}[M]\left(\frac{\phi\varepsilon[PI]I_0 e^{-\varepsilon[PI]z}\lambda}{N_A ch*10^3}\right)^{\frac{1}{2}} \quad (3)$$

where $k_{eq} = \frac{k_p}{\sqrt{k_t}}$. Combination of Eq. (1) and (3) yields

$$-\frac{d[M]}{[M]} = k_{eq}\left(\frac{\phi\varepsilon[PI]I_0 e^{-\varepsilon[PI]z}\lambda}{N_A ch*10^3}\right)^{\frac{1}{2}} dt \quad (4)$$

which on integration leads to

$$ln\frac{[M]}{[M]_0} = -k_{eq}\left(\frac{\phi\varepsilon[PI]I_0 e^{-\varepsilon[PI]z}\lambda}{N_A ch*10^3}\right)^{\frac{1}{2}} t \quad (5)$$

where $[M]_0$ is the initial concentration of monomer and $[M]$ is the concentration of monomer at time t. The polymerized fraction can be expressed as

$$Polymerized\ fraction = \frac{[M]_0-[M]}{[M]_0} = \left(1-\frac{[M]}{[M]_0}\right) \quad (6)$$

which can be combined with Eq. (5) to yield

$$Polymerized\ fraction = [1 - exp(-k_{eq}\left(\frac{\phi\varepsilon[PI]I_0e^{-\varepsilon[PI]z}\lambda}{N_Ach*10^3}\right)^{\frac{1}{2}} t)] \tag{7}$$

The PI efficiency, ϕ, of TPO is estimated to be 0.6 in the simulations [2]. In the main text, we present this equation as

$$Polymerized\ fraction = [1 - exp(-k_{eq}\left(\beta\varepsilon[PI]I_0e^{-\varepsilon[PI]z}\right)^{\frac{1}{2}}t)] \tag{7b}$$

with $\beta = \phi\lambda/(N_Ach \cdot 10^{-3})$.

Calculation of the exposure time scale as a function of the Weber number

The flow rate of a liquid jet through a nozzle can be expressed as

$$Q = V\pi\frac{D_j^2}{4} \tag{8}$$

where V is the velocity of jet, D_j is the diameter of the jet which is assumed to be equal to the nozzle. Depending on viscosity and interaction with the nozzle, the jets are not always exactly same as nozzle diameter. This assumption is made since no obvious difference between the diameter of liquid jet and the nozzle diameter is observed. The Weber number of the liquid jet represents the ratio between inertial energy and surface energy, and can be expressed as

$$We = \frac{\rho V^2 D_j}{\sigma} \tag{9}$$

Where ρ and σ are the density and surface tension of the ejected liquid. Combination of Eq. (8) and (9) yields

$$D_j = \left(\rho * \frac{16*Q^2}{We.\sigma\pi^2}\right)^{\frac{1}{3}} \tag{10}$$

The exposure time of the jet to the irradiation window can be expressed as

$$\tau = \frac{l}{V} \tag{11}$$

where $l = 8 \times 10^{-3}\ m$ is the exposure distance and is equivalent to the height of the irradiation window. Combination of Eq. (11) and (8) yields

$$\tau = \frac{l\pi D_j^2}{4Q} \tag{12}$$

Combination of Eqs. (10) and (12) yields the timescale of reaction as a function of Q

$$\tau = l\left(\frac{Q}{\pi}\right)^{\frac{1}{3}}\left(\rho * \frac{2}{We.\sigma}\right)^{\frac{2}{3}} \tag{13}$$

Calculation of bending radius of Janus fibers

The Timoshenko model [3] is applied to calculate the bending radius, which is a general formulation that can be used for bilayer bending arising from different types of strain [4]. The curvature of Janus fiber is modeled as two connected plates with different swelling ratio, Young's modulus, and stiffness,

$$\kappa = \frac{\alpha_1 - \alpha_2}{h_2}\frac{6mn(1+m)}{1+4mn+6m^2+4m^3n+m^4n^2} \tag{14}$$

with

$$m = \frac{h_1}{h_2} \tag{15}$$

$$n = \frac{E_1}{E_2} \tag{16}$$

where α_1 and α_2 are the actuation strain of the active layer (PEGDA in our case) and passive layer (epoxy), h_1 and h_2 are the thickness of PEGDA layer and epoxy layer, E_1 and E_2 are the Young's modulus of PEGDA layer and epoxy layer. α_1 and α_2 were obtained by measuring the strain of the two materials after swelling (Figure S13). h_1 and h_2 were measured under a microscope while E_1 and E_2 were calculated from the strain stress curve (Figure S12).

In our experiments, the total thickness of PEGDA and epoxy, *H*, was found to be relatively constant (3.83 ± 0.35 ×10^{-4} m). The h_2 can be expressed as a function of *m*

$$h_2 = \frac{\mathrm{H}}{1+m} \tag{17}$$

Combine Eqs. (14) and (17) yields

$$\kappa = \frac{(1+m)(\alpha_1-\alpha_2)}{\mathrm{H}} \frac{6mn(1+m)}{1+4mn+6m^2+4m^3n+m^4n^2} \tag{18}$$

The calculated average value of κ was obtained when H was set as 3.83 ×10^{-4} m and calculated upper or lower boundary value of κ was obtained when H was set as 3.48 ×10^{-4} m or 4.18 ×10^{-4} m, respectively.

Supporting Figures

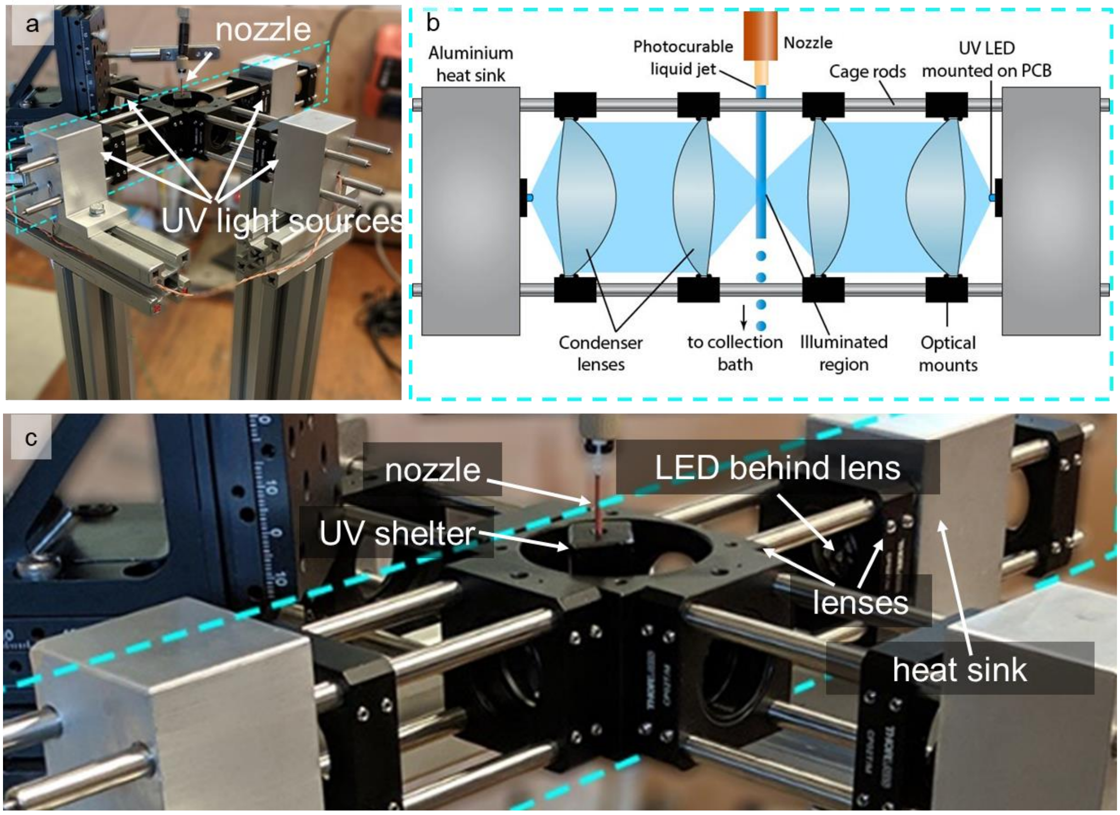

Figure S1. Picture and scheme of the jet curing setup. (a)Picture of main part of the setup. (b) Scheme showing the composition of the light source. (c) Picture detailing the main components of the setup.

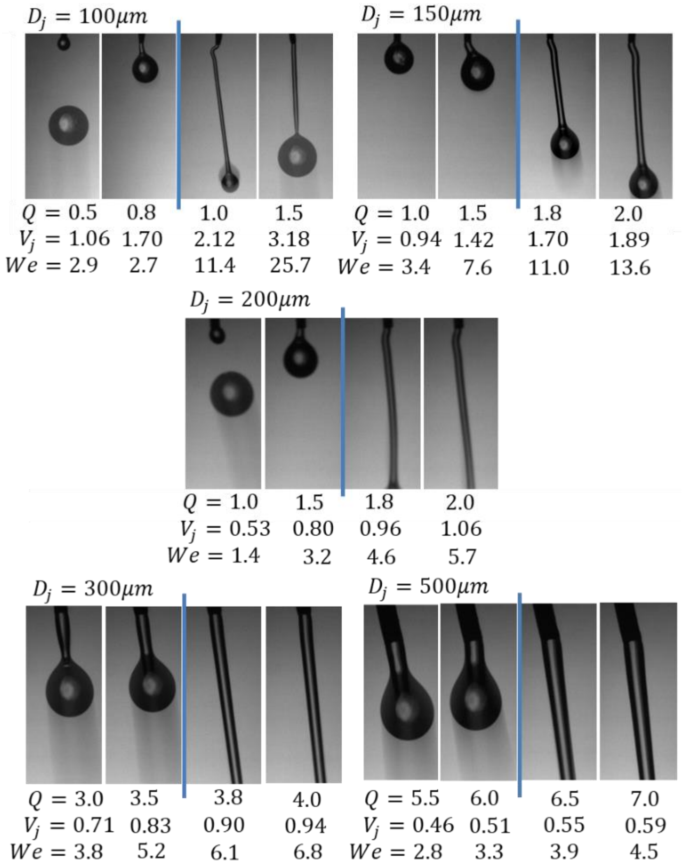

Figure S2. Still images from the videos showing the dripping-jetting transitions of nozzles with varying diameter, D_j. The flow rate, *Q*, is indicated in ml/min and jet velocity, V_j, is in m/s. Pure PEGDA was used as the testing ink. Blue lines indicate the transition from dripping regime to jetting regime. Typical experiments for fiber production were performed at higher Weber number (up to ~20 for 100 µm nozzle), as indicated in Figure 2 but not shown here.

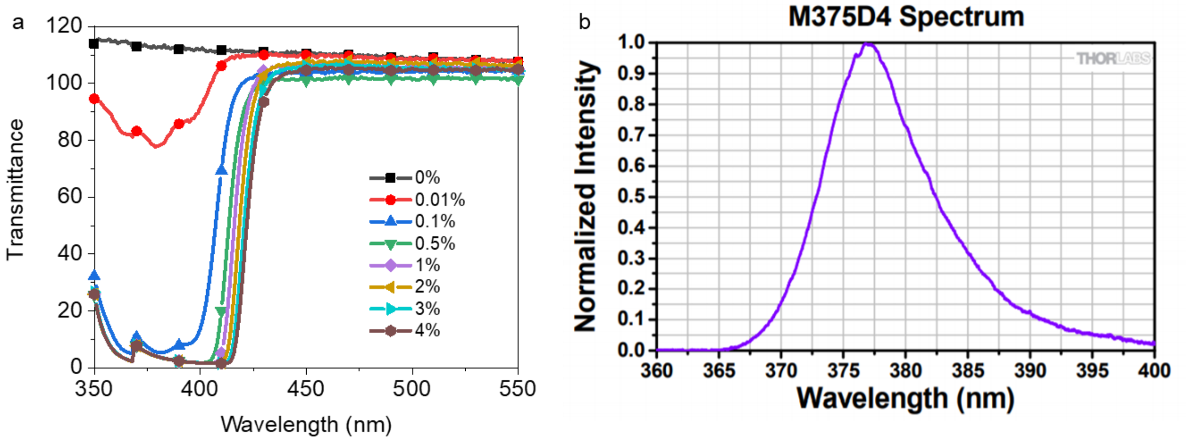

Figure S3. Match the absorption wavelength of the photoinitiator (PI) with the emitting wavelength of the light source. (a) UV-Vis spectroscopy shows transmittance of the PI, indicates strong absorption from 370 nm to 410 nm. The PI was dissolved into ethanol to record transmittance; ethanol was used as the reference (0%). The length of the light path through the sample is 1 cm. (b) The emission peak of the LED light source appears between 370 nm to 390 nm, being fully covered by the absorbing range of the PI. Data copied from Thorlabs spec sheet of the LED product (M375D4).

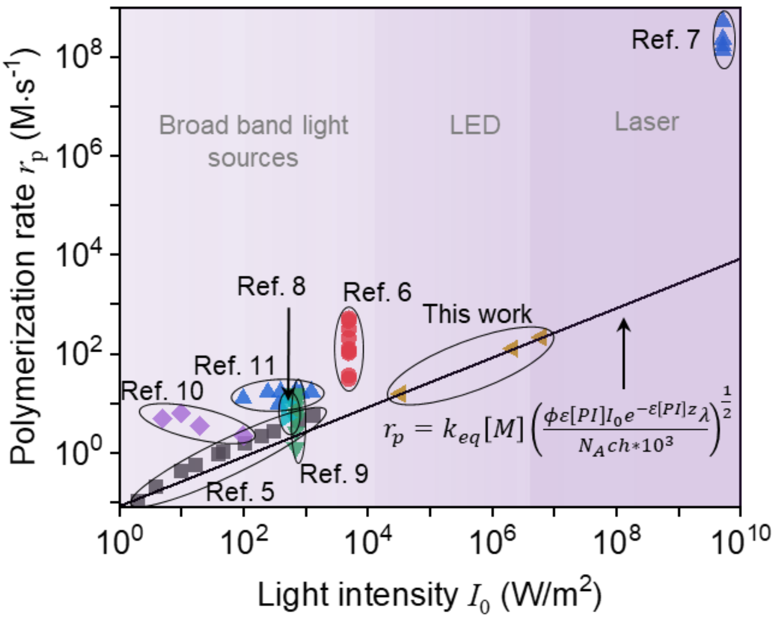

Figure S4. Comparison of our calculated polymerization rate r_p with reported values [5-11]. The r_p values of the three data points shown for "This work" from left to right are calculate from I_0 as 34000 W/m^2 (4 LEDs focused to 8 mm × 8 mm square), 2176000 W/m^2 (4 LEDs focused to 1 mm × 1 mm square), and 6250000 W/m^2 (laser diode), respectively.

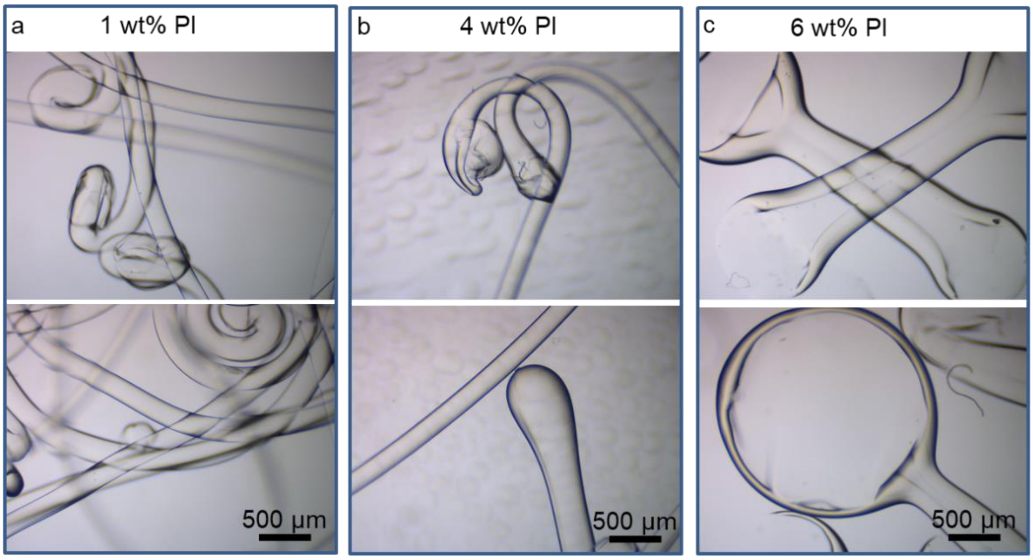

Figure S5. Microscope images of fibers produced at varying concentrations of PI. (**A**) The ends of fibers curled severely when the concentration of PI was 0.029 M (1 wt%). (**B**) Fibers with relatively straight ends were observed when PI concentration was 0.115 M (4 wt%). The drop-like shapes on the background might be uncured ink that was collected when the UV light was off or contaminations on the outer surface of the petri dish. (**C**) The ink became very viscous after preparation when PI concentration was 0.172 M (6 wt%), which makes the jet unstable, and only irregular fiber ends were obtained. For all the experiments, 150 µm nozzles were used and a small amount (2 v/v%) of ethanol was added to facilitate the dissolving of PI.

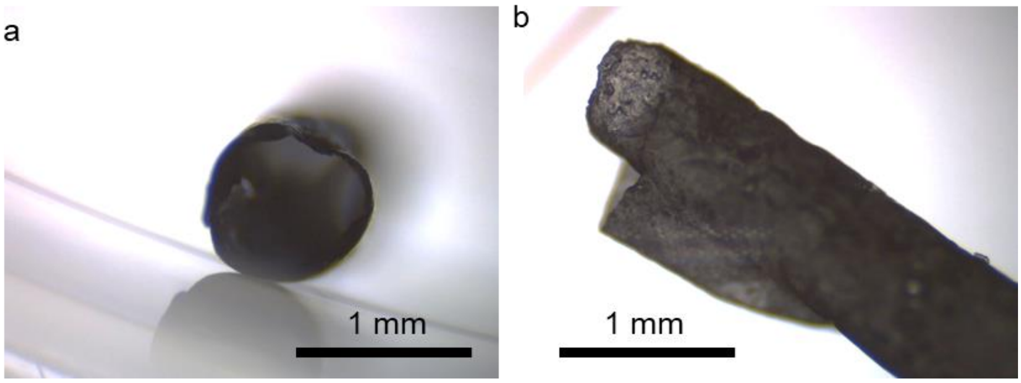

Figure S6. Microscope images of hollow fibers produced by a homogeneous thick liquid jet. The monomer jet is ejected from a tubing with 1.27 mm diameter (radius of 0.63 mm) and the concentration of photoinitiator is 6%.

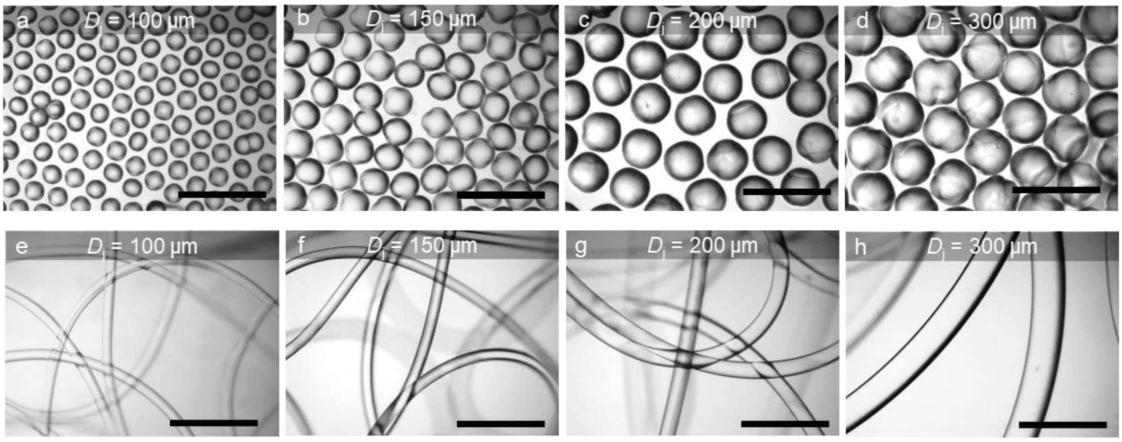

Figure S7. PEGDA Microparticles and microfibers with controllable size. Scale bars represent 1 mm.

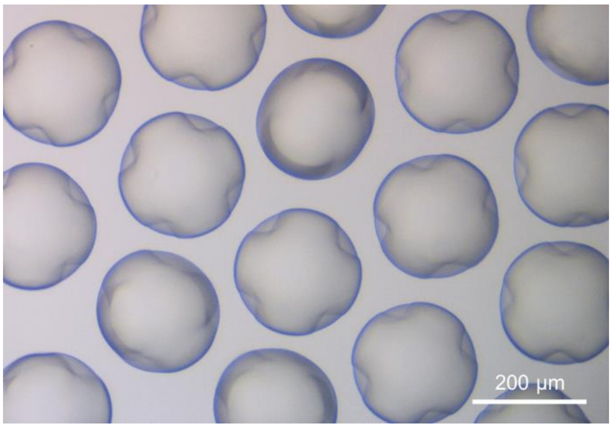

Figure S8. Optical microscope image showing the PEGDA particles made with 100 µm nozzle in detail. The particles have cross-shaped inner structures although the outer shape is virtually circular (as revealed by the particle in the top-middle, which is oriented "vertically"). The heterogeneous structure might be a result of varying polymerization degree of PEGDA inside the particle generated by non-even exposure to the four-directional UV light. It is expected the non-uniformity can be prevented by adjusting the number or type of UV light sources.

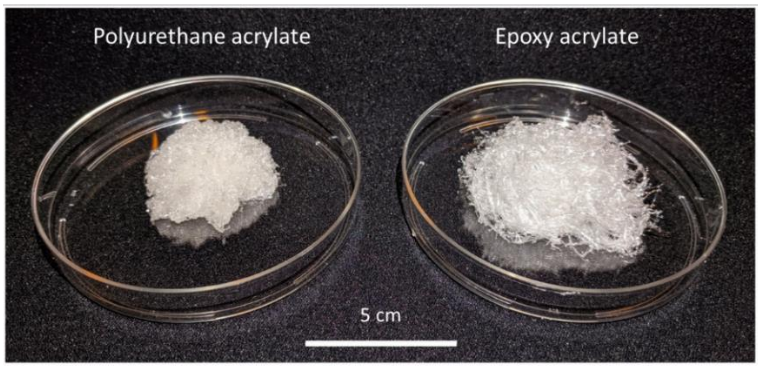

Figure S9. High throughput production of Polyurethane acrylate and epoxy acrylate fibers. Those two samples composed of around 15000 short fibers produced in 2.5 minutes at 100 fibers/second. The fiber morphology of both samples is similar, but the stiffer epoxy-enhanced resin appears larger since the fibers hardly bend.

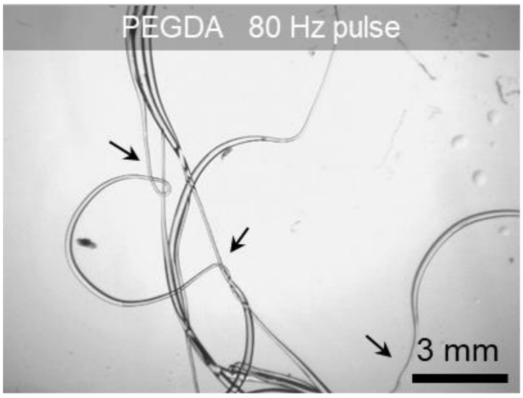

Figure S10. Partially connected short fiber of PEGDA obtained at the pulsing frequency of 80Hz. Partial connections are indicated by arrows.

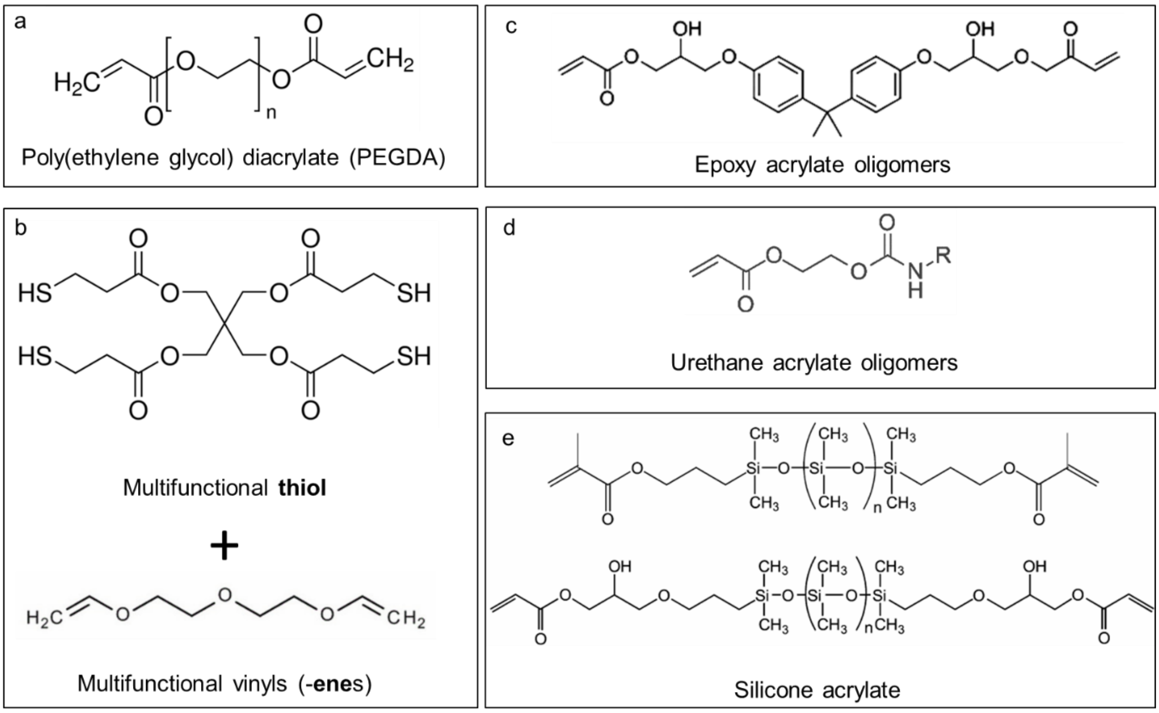

Figure S11. Molecular structure of varying photocrosslinkable monomers (oligomers) used in this work.

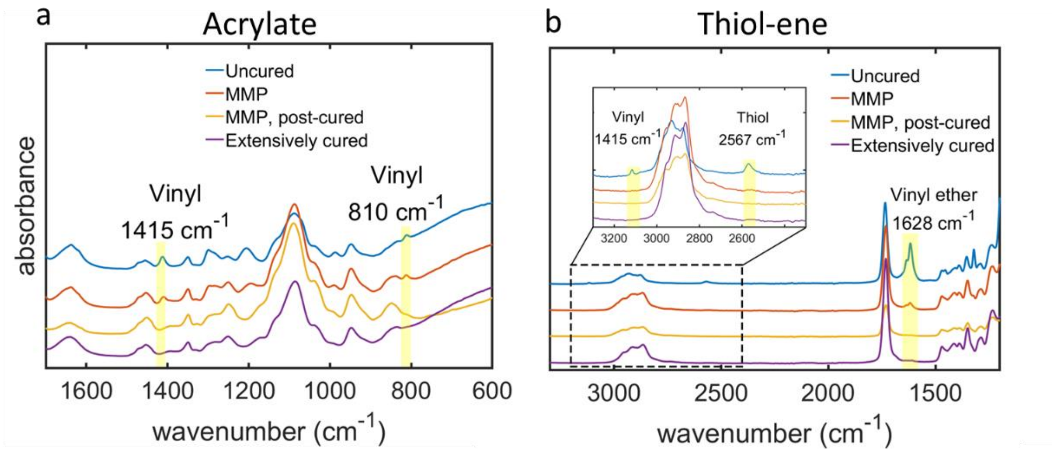

Figure S12. FTIR spectroscopy of PEGDA (a) and thiol-ene (b) monodisperse microparticle (MMP) and controls confirming the formation of crosslinking in the produced particles. Functional peaks to assess crosslinking are highlighted in yellow.

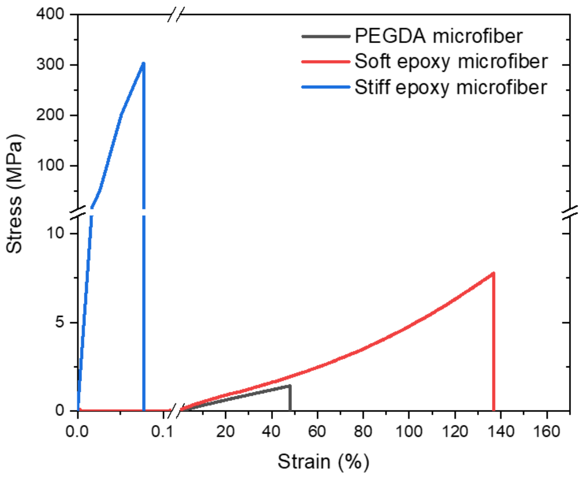

Figure S13. Mechanical performance of PEGDA microfiber and epoxy microfibers. Epoxy fibers with varying tensile stress from 10 MPa to 300 MPa were produced. Stiff epoxy fiber as produced by focusing the UV light into ~1 mm × 1 mm square to increase the UV dose.

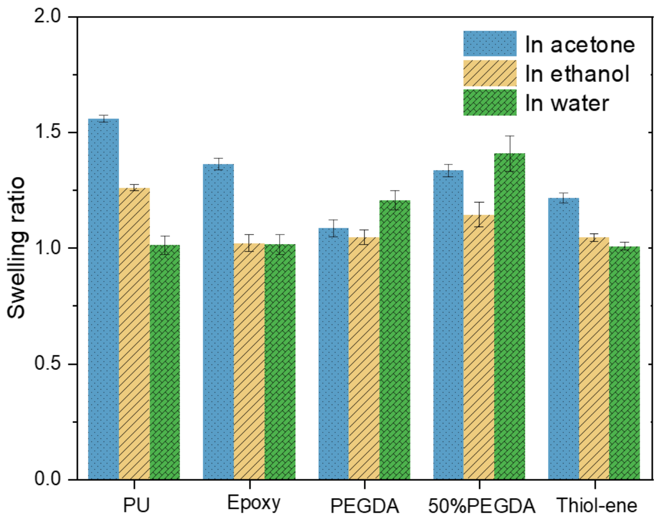

Figure S14. The swelling ratio of varying polymer to water and solvents. PEGDA shows 1.2 swelling ratio to water while Epoxy almost has no swelling effect to water. PU represents polyurethane.

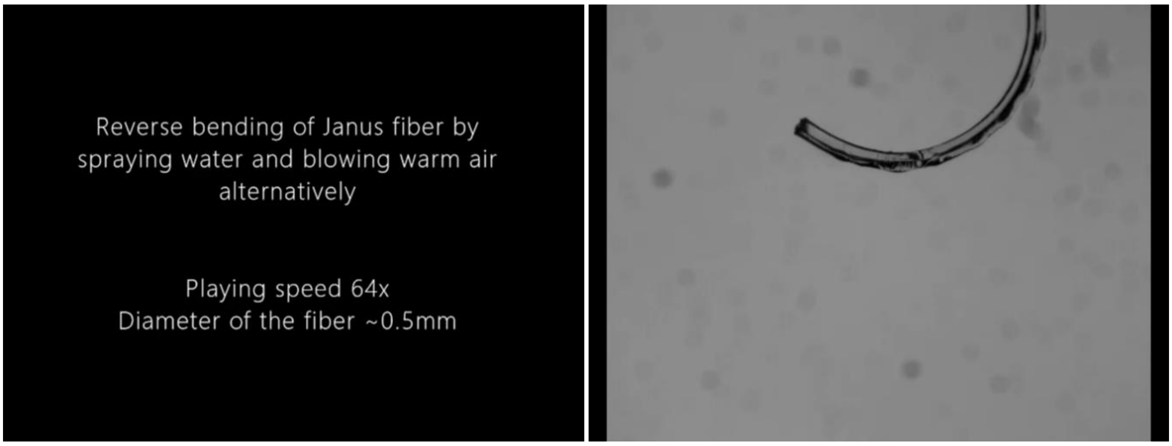

Movie S1. Reversible bending of the Janus fiber. The bending was triggered by spraying water to the fiber. Warm air was blown to the wetted fiber, allowing it to be dried quickly. The structure of the responsive fiber kept stable after ~40 reversible bending cycles.

[1] G. Odian, in *Principles of polymerization*, John Wiley & Sons, **2004**, 206.
[2] S. Jockusch, I. V. Koptyug, P. F. McGarry, G. W. Sluggett, N. J. Turro, D. M. Watkins, *J. Am. Chem. Soc.* **1997**, *119*, 11495.
[3] S. Timoshenko, *Josa* **1925**, *11*, 233.
[4] M. Christophersen, B. Shapiro, E. Smela, *Sensors Actuators B: Chem.* **2006**, *115*, 596.
[5] P. D. Iedema, V. Schambock, H. Boonen, J. Koskamp, S. Schellekens, R. Willemse, *Chem. Eng. Sci.* **2018**, *176*, 491.
[6] C. Decker, K. Moussa, *ACS Symp. Ser.* **1990**, *417*, 439.
[7] C. Decker, *J. Polym. Sci. A Polym. Chem.* **1983**, *21*, 2451.
[8] C. Decker, B. Elzaouk, D. Decker, *J. Macromol. Sci. Pure Appl. Chem.* **1996**, *A33*, 173.
[9] C. Decker, K. Moussa, *Macromolecules* **1989**, *22*, 4455.
[10] N. B. Cramer, S. K. Reddy, A. K. O'Brien, C. N. Bowman, *Macromolecules* **2003**, *36*, 7964.
[11] C. Decker, D. Decker, *J. Macromol. Sci. Pure Appl. Chem.* **1997**, *A34*, 605.
